# Balloon-Assisted Enteroscopy for Retrieval of Small Intestinal Foreign Bodies: A KASID Multicenter Study

**DOI:** 10.1155/2020/3814267

**Published:** 2020-05-19

**Authors:** Jeongseok Kim, Beom Jae Lee, Nam Seok Ham, Eun Hye Oh, Kee Don Choi, Byong Duk Ye, Jeong-Sik Byeon, Chang Soo Eun, Jin Su Kim, Dong-Hoon Yang

**Affiliations:** ^1^Department of Gastroenterology, University of Ulsan College of Medicine, Asan Medical Center, Seoul, Republic of Korea; ^2^Department of Internal Medicine, Keimyung University School of Medicine, Daegu, Republic of Korea; ^3^Department of Gastroenterology, Korea University Guro Hospital, Seoul, Republic of Korea; ^4^Department of Gastroenterology, Veterans Health Service Medical Center, Seoul, Republic of Korea; ^5^Department of Gastroenterology, Haeundae Paik Hospital, Inje University College of Medicine, Busan, Republic of Korea; ^6^Department of Internal Medicine, Hanyang University Guri Hospital, Guri, Republic of Korea; ^7^Division of Gastroenterology, Department of Internal Medicine, Seoul St. Mary's Hospital, College of Medicine, The Catholic University of Korea, Seoul, Republic of Korea

## Abstract

**Background and Aims:**

Balloon-assisted enteroscopy (BAE) can be used to retrieve small intestinal foreign bodies (FBs). Here, we aimed at exploring the clinical usefulness of BAE for the retrieval of small intestinal FBs.

**Methods:**

We retrospectively reviewed the medical records of 34 patients who underwent BAE to retrieve small intestinal FBs at 3 tertiary referral centers between April 2005 and June 2017.

**Results:**

The retained materials included capsule endoscopes (CEs; *n* = 18 [52.9%]), self-expandable metal stents (SEMSs; *n* = 5 [14.7%]), biliary drainage catheters (*n* = 4 [11.8%]), gallstones (*n* = 3 [8.8%]), an embolization coil (2.9%), a needle, an intragastric bariatric balloon, and a razor blade. FBs were located or stuck in the ileum (*n* = 17 [50%]), jejunum (*n* = 16 [47.1%]), and an undetermined small intestinal segment (*n* = 1). Seventeen cases of FBs (50%; 7 CEs, 3 biliary drainage catheters, 3 SEMSs, 2 gallstones, 1 intragastric balloon, and 1 needle) were successfully retrieved enteroscopically. FBs of 4 asymptomatic patients (3 CEs and 1 razor blade) passed spontaneously. The remaining 13 patients underwent surgery for persistent or symptomatic FBs: 12 were successfully removed and 1 CE removal procedure failed due to severe peritoneal adhesions. The presence of symptoms was the only independent predictor of successful retrieval using BAE (odds ratio 13.40, 95% confidence interval 1.10–162.56, *P* = 0.042). BAE-related complications such as bowel perforation and acute pancreatitis occurred in 2 patients (5.9%).

**Conclusions:**

BAE can be the first option for FB removal in the small intestine. The presence of symptoms was associated with successful enteroscopic retrieval.

## 1. Introduction

The retention of foreign bodies (FBs) in the small intestine can occur after their ingestion by patients who have a normal small intestine or an underlying intestinal pathology such as stricture, adhesion, mass, or diverticulum [1]. Surgical treatment was historically the first option for FB retrieval when patients presented with symptoms related to complications such as intestinal obstruction, perforation, and bleeding [2]. Since the introduction of balloon-assisted enteroscopy (BAE) including double-balloon enteroscopy (DBE) and single-balloon enteroscopy (SBE), surgery is occasionally used for FB retrieval from the small intestine [2–8].

According to the clinical guideline of European Society of Gastrointestinal Endoscopy, batteries, magnets, sharp-point FBs, large FBs > 5–6 cm in diameter, and food boluses in the stomach or small intestine require urgent endoscopic removal (within 24 hours), while blunt and small- or medium-sized FBs require nonurgent (within 72 hours) removal [9]. For the removal of small intestinal FBs using BAE, the systemic condition of the patient, availability of accessories, procedure time, and route of scope insertion should be considered [10]. Although several case series and studies have reported FB retrieval using BAE, consensus guidelines are lacking [11–15].

Here, we aimed at exploring the clinical usefulness and safety of BAE for the retrieval of various small intestinal FBs and evaluating the factors associated with retrieval success.

## 2. Methods

### 2.1. Patients

We reviewed the medical records of patients who underwent BAE for the retrieval of small intestinal FBs at 3 tertiary referral centers between April 2005 and June 2017. The patients' demographic and clinical data were retrospectively collected. The study protocol was approved by the institutional review boards of each center.

### 2.2. BAE Procedures

Before the BAE, abdominal radiography and computed tomography (CT) were performed to identify the presence and location of the FBs. Based on the CT finding, the route of entry was determined. Either SBE (SIF-Q180 enteroscope; Olympus Medical Inc., Tokyo, Japan) or DBE (EN-450-T5 or EN-580T enteroscope; Fujinon Inc., Saitama, Japan) was used for FB removal according to the availability of equipment at each institution. Details of the insertion technique for SBE or DBE were previously described [16]. In the case of antegrade (per oral), BAE was performed after fasting at least 6 hours; and in the case of retrograde (per anal), BAE was performed after bowel cleansing with polyethylene glycol solution. The scope was advanced as deep as possible or until FBs were encountered. In the case of small intestinal strictures, balloon dilation was attempted before passing the stenotic site to retrieve FBs. All procedures were performed by experienced board-certified endoscopists under conscious sedation with cardiorespiratory monitoring.

### 2.3. Variables and Outcomes

Patient-related variables, such as symptoms and signs related to small intestinal FBs, coexistent small bowel diseases, previous history of abdominal surgery, and date of FB ingestion, were collected from the medical records. The types and characteristics of the ingested FBs were reviewed. FB size was measured upon removal. In cases of retrieval failure, size was digitally measured on the radiographic images. FB location in the small bowel was estimated based on the CT results. BAE insertion route, procedure time, and balloon dilation for stricture were investigated. The primary outcome was the enteroscopic FB retrieval. Spontaneous evacuation or surgical removal of FBs was verified in cases of enteroscopic retrieval failure. Procedure-related complications such as perforation, bleeding, and pancreatitis were also recorded.

### 2.4. Statistical Analysis

The Mann-Whitney *U*-test was used to compare continuous data. Categorical data were analyzed using Pearson's Chi-square test or Fisher's exact test. Univariable logistic regression analyses were performed to estimate the odds ratios (ORs) for the successful retrieval of all FBs using BAE. Age, sex, and variables with *P* values < 0.2 from univariable analyses were included in the multivariable analysis with the enter method. In the same way, univariable analyses were performed to estimate the ORs for successful retrieval of capsule endoscopes (CEs); however, the multivariable analysis could not be performed due to the small sample size and small number of events per predictor parameter. The data were analyzed using SPSS for Windows (version 21.0; SPSS, Chicago, IL, USA). Two-sided *P* values of less than 0.05 were considered statistically significant.

## 3. Results

### 3.1. Characteristics of Patients and FBs

A total of 34 patients underwent SBE or DBE for the retrieval of small intestinal FBs during the study period. The median patient age was 57 years (range, 19–92 years), and 23 patients were male (67.6%). Sixteen patients (47.1%) had a prior history of abdominal or pelvic surgery. Small intestinal diseases causing FB retention were identified by radiological evaluation, capsule endoscopy, BAE, or surgery in 15 patients (see Table 1). CEs (*n* = 18 [52.9%]) were the most frequently observed retained objects. Twenty patients (58.8%) complained of obstructive symptoms such as abdominal pain, nausea, vomiting, and abdominal distension, while 10 patients (29.4%) showed obstructive signs such as ileus on abdominopelvic CT scans. The date of FB retention was available for 32 patients (94.1%), and the median interval between FB retention and BAE was 10 days (range, 1–364 days).

### 3.2. BAE Outcomes and Safety

**Figure 1:**
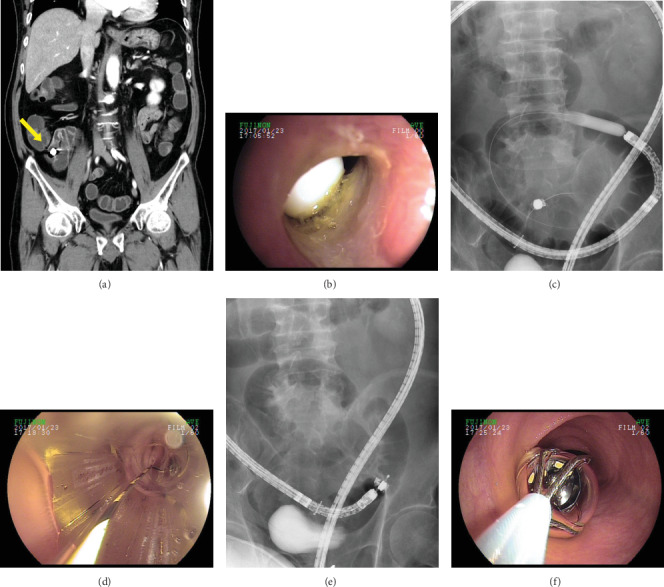
Successful retrieval of a capsule endoscope (CE) using a basket. The CE was located at the ileum on the abdominopelvic computed tomography image (yellow arrow) (a). Fluoroscopy-guided double-balloon endoscopy (DBE) showing that the CE was impacted by stenosing ulceration associated with nonsteroidal anti-inflammatory drug use approximately 60 cm proximal to the ileocecal valve (b). Enteroscopic balloon dilation was performed using a through-the-scope balloon dilation catheter (CRE™ balloon catheter; Boston Scientific, Natick, MA, USA) (c, d). After successful DBE-assisted balloon dilation, the capsule endoscope was retrieved using a basket (e, f).

The median BAE procedure time was 75 min (range, 20–250 min). Anterograde, retrograde, and bidirectional approaches were performed in 21 (61.8%), 10 (29.4%), and 3 (8.8%) patients, respectively, using SBE (*n* = 8 [23.5%]) or DBE (*n* = 26 [76.5%]). Balloon dilatation at stricture sites was performed in 6 patients (17.6%). FBs were successfully retrieved by BAE in half of the patients (*n* = 17). A case of successful CE retrieval is depicted in Figure 1. Procedure-related complications occurred in 2 patients. One patient underwent surgery due to bowel perforation during balloon dilation for multiple small bowel strictures, while the other developed pancreatitis after the procedure that recovered by conservative management.

### 3.3. Factors Associated with Enteroscopic FB Retrieval Success

The presence of symptoms was the only factor associated with the successful retrieval of all FBs and CEs (both *P* = 0.013, see Table 2). Of total patients, the presence of obstructive signs showed marginal significance (*P* = 0.057), while FB location (*P* = 0.094) was not significantly associated with successful retrieval. In multivariable analysis, the presence of symptoms was the only independent predictor of successful retrieval of all FBs (OR 13.40, 95% confidence interval (CI) 1.10-162.56, *P* = 0.042, see Table 3). Among 18 patients with CE retention, all variables except for the presence of symptoms did not show a significant association (see Tables 2 and 4).

### 3.4. Management and Clinical Outcomes of Enteroscopic FB Retrieval Failure

**Figure 2:**
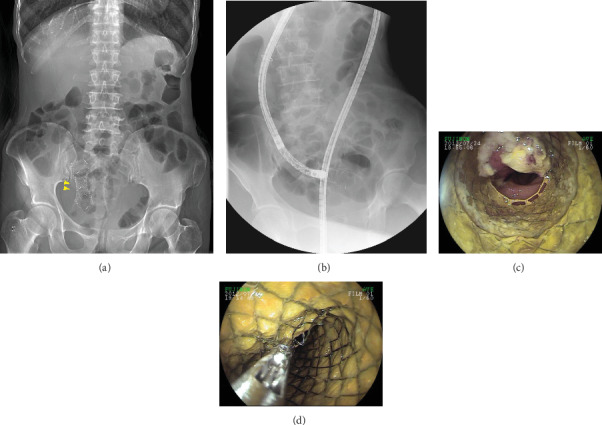
Retrieval failure of a self-expandable metal stent (SEMS) using rat tooth forceps. The SEMS was impacted in the ileum on an abdominal X-ray image (yellow arrowheads) (a). Fluoroscopy-guided double-balloon endoscopy showing that the SEMS was impacted at about 15 cm proximal to the ileocecal valve (b). The SEMS could not be retrieved using rat tooth forceps because of the severe intestinal edema surrounding it (c, d).

Among the 17 patients in whom retrieval failed, 14 had previous history of abdominal surgery (*n* = 9), Crohn's disease (*n* = 5), small-bowel malignancy (*n* = 2), and nonsteroidal anti-inflammatory drug- (NSAID-) induced enteropathy (*n* = 2). The FB locations (11 CEs, 1 self-expandable metal stent (SEMS), and 1 embolization coil) could not be reached due to intestinal strictures and/or abdominal adhesions. Although the impacted location could be reached in the remaining 4 patients (razor blade, gallstone, biliary drainage catheter, and SEMS), they could not be retrieved by BAE. The razor blade in a patient who did not have small intestinal pathology migrated to the distal part of the small intestine during the retrieval procedure. An impacted 2 cm cholesterol gallstone was found in a patient who underwent open cholecystectomy and choledochojejunostomy due to biliary stones 30 years prior. DBE reached the impacted location but failed to remove gallstones due to obstructive cancer at the choledochojejunal anastomosis site. A biliary drainage catheter in a patient with recurrent cancer could not be retrieved using rat tooth forceps due to tumor invasion. Finally, SEMS in a patient with a previous history of total gastrectomy with esophagojejunostomy could not be retrieved by rat tooth forceps because of severe intestinal edema around the impacted area (see Figure 2).

Thirteen patients underwent surgery, of whom FBs were successfully retrieved in 12 (7 CEs, 2 SEMSs, a biliary drainage catheter, a gallstone, and an embolization coil), while CE removal failed in 1 due to intestinal adhesions caused by peritoneal seeding of common bile duct cancer. In the remaining 4 patients with stricturing Crohn's disease (*n* = 1), previous abdominal surgery (*n* = 1), and no specific underlying disease (*n* = 2), 3 CEs and 1 razor were spontaneously excreted.  Table 1 summarizes the characteristics of the successful and failed FB retrievals.

## 4. Discussion

In the present study, FBs impacted in the small intestine were successfully retrieved using BAE in half of the patients; 2 patients (5.9%) experienced procedure-related complications. The presence of entrapment-related symptoms was the only significant predictor of successful retrieval.

About 80–90% of FBs including food bolus ingested in the gastrointestinal tract passed spontaneously without complications [17]. It generally takes about 4–6 days, rarely up to 4 weeks, if they traverse the esophagus [9]. Of them, 10–20% require endoscopic removal, and 1% or less require surgery [17]. In contrast, in cases of intentional ingestion, the rates of endoscopic intervention (63–76%) and surgery (12–16%) were reportedly much higher [18, 19]. In the present study, all patients underwent BAE with the following indications: entrapment-related symptoms (*n* = 20) or signs (*n* = 10), sharp-point FBs (*n* = 2), or entrapment duration (*n* = 10). Thirteen of 17 patients with FBs underwent surgery.

Although there are few guidelines related to the management of ingested FBs in the upper gastrointestinal tract, guidelines on the retrieval of entrapped FBs in the small intestine are lacking [9, 10]. Various entrapped FBs such as CEs, bezoars, metal stents, plastic forks, dental reamers, nails, medical tubes, coins, bones, root canal needles, cellophane wall of a patency capsule, and press-through packages of medicine were successfully removed using BAE in previous anecdotal case reports and studies with small sample sizes [2, 3, 5, 13–15, 20–27].

In the present study, most of the FBs entrapped within the small intestine were medical devices (29/34 [85.3%]), and the incidental ingestion of razor blades and needles were observed in 2 patients. Our results showed that successful FB retrieval was only significantly associated with entrapment-related symptoms (82.4% vs. 35.3%, *P* = 0.013) among various clinical characteristics. Furthermore, it was the only independent predictor of successful retrieval of all FBs (OR 13.40, 95% CI 1.10-162.56, *P* = 0.042). Obstructive sign was slightly more prevalent in patients with successful retrieval (47.1% vs. 11.8%, *P* = 0.057). Our data cannot directly explain why successful FB retrieval was associated with entrapment-related symptoms in small intestinal FB cases. However, given that FBs located in ileum were more common in asymptomatic patients than in symptomatic patients (75.0% vs. 36.4%, *P* = 0.020), FBs not causing obstructive symptoms were likely to migrate toward the more distal part of the small bowel where BAE could not approach. On the other hand, FBs causing obstructive symptoms and signs were probably fixed in the original location of entrapment without distal migration until enteroscopic retrieval was attempted. Therefore, we suggest that FB retrieval using BAE can be considered the first option for patients with obstructive symptoms or signs but without peritoneal irritation signs. Moreover, among 9 patients without entrapment-related symptoms and signs in whom FB retrieval failed, 4 (3 CEs, 1 razor blade) experienced spontaneous passage. This suggests that watchful waiting without undergoing BAE is possible if entrapment-related symptoms and signs are absent. Although the lumen diameter of the stricture segment at the small intestine was not measured in the present study, the frequency of spontaneous passage was significantly lower in patients with a smaller lumen diameter at the stricture site (less than two-thirds the capsule diameter) (*P* = 0.004) [28]. Meanwhile, considering that the impacted location could not be reached in 13 patients, it should be recognized that the risk of FB retrieval failure will be high in patients with suspicious abdominal adhesions or intestinal strictures.

CEs were the most common FBs entrapped in the small intestine (*n* = 18), with a retention rate of 0.3–13% among populations [1, 28–32]. In a Korean nationwide study, the underlying diseases of the small intestine causing CE retention (32/1,291 [2.5%]) include Crohn's disease (16/32 [50%]), malignant tumor (4/32 [12.5%]), intestinal tuberculosis (3/32 [9.4%]), postoperative benign stricture (2/32 [6.3%]), and NSAID-induced stricture (1/32 [3.1%]) [28]. In a study conducted at the Mayo Clinic, NSAID-induced enteropathy was the most common cause (11/14 [78.6%]) of capsule retention, but referral bias might have influenced the study results [1, 12]. Similarly, CE (18/34 [52.9%]) was the most common FB requiring removal using BAE in the present study, and the major causes accompanying CE retention were Crohn's disease (*n* = 7), NSAID-induced enteropathy (*n* = 3), and postoperative benign stricture (*n* = 2). Surgery was historically considered the first-line treatment for removing retained CEs, but BAE is currently advocated as the primary method despite limited data [1, 10, 33]. The success rate of CE retrieval using BAE was 38.9% (7/18) in the present study, which was relatively lower than the previous reports (range, 56–91.7%) [11, 12, 29, 34]. We think that it was related to the high proportion of the ileal location (13/18 [72.2%]). In a retrospective analysis of the largest number of patients with capsule retention (*n* = 44), successful retrieval by DBE were significantly associated with anterograde route (*P* < 0.001), jejunal or proximal location (*P* = 0.013), and 3 or fewer strictures (*P* = 0.049) [34].

This study was limited by its retrospective design and small number of patients in 3 medical institutions. In addition, various FBs, underlying diseases, and surgical histories may interfere with the generalizability of our findings. However, since the actual frequency of FB removal using BAE is not high in many institutions, this study may provide useful clinical information.

## 5. Conclusion

BAE is a safe method that can be considered the first-line option for removing FBs impacted in the small intestine, and the probability of retrieval success is significantly higher in patients with entrapment-related symptoms. Further prospective studies with large patient number are warranted to generalize our findings.

## Figures and Tables

**Table 1:** Clinical characteristics according to foreign body retrieval results using balloon-assisted enteroscopy.

Variables	Retrieval success (*n* = 17)	Retrieval failure (*n* = 17)	Total (*n* = 34)
Age (yr), median (range)	62 (32-92)	55 (19-86)	57 (19-92)
Male (*n* (%))	11 (64.7%)	12 (70.6%)	23 (67.6%)
Presence of small bowel-involved disease (*n* (%))			
Crohn's disease	3 (17.6%)	5 (29.4%)	8 (23.5%)
Small-bowel malignancy	2 (11.8%)	2 (11.8%)	4 (11.8%)
NSAID-induced enteropathy	1 (5.9%)	2 (11.8%)	3 (8.8%)
Previous abdominal surgery (*n* (%))	7 (41.2%)	9 (52.9%)	16 (47.1%)
Roux-en-Y hepaticojejunostomy	1 (5.9%)	1 (5.9%)	2 (5.9%)
Pylorus-preserving Whipple	1 (5.9%)	2 (11.8%)	3 (8.8%)
Total gastrectomy with esophagojejunostomy	1 (5.9%)	1 (5.9%)	2 (5.9%)
Right hemicolectomy	2 (11.8%)	0	2 (5.9%)
Appendectomy	0	2 (11.8%)	2 (5.9%)
Small-bowel resection and anastomosis	0	2 (11.8%)	2 (5.9%)
Hepatectomy, cholecystojejunal anastomosis	0	1 (5.9%)	1 (2.9%)
Nephrectomy	1 (5.9%)	0	1 (2.9%)
Cholecystectomy	1 (5.9%)	0	1 (2.9%)
Retained material (*n* (%))			
Capsule endoscope	7 (41.2%)	11 (64.7%)	18 (52.9%)
Self-expandable metal stent	3 (17.6%)	2 (11.8%)	5 (14.7%)
Biliary drainage catheter	3 (17.6%)	1 (5.9%)	4 (11.8%)
Gallstone	2 (11.8%)	1 (5.9%)	3 (8.8%)
Embolization coil	0	1 (5.9%)	1 (2.9%)
Needle	1 (5.9%)	0	1 (2.9%)
Razor blade	0	1 (5.9%)	1 (2.9%)
Intragastric bariatric balloon	1 (5.9%)	0	1 (2.9%)
Size of retained material (mm), median (range)			
Length	26 (10-120)	26 (20-220)	26 (10-220)
Diameter	11 (1-50)	11 (1-20)	11 (1-50)
Duration of retention (days), median (range)	8 (1-23)	15 (0-364)	10 (0-364)
Presence of symptoms (*n* (%))	14 (82.4%)	6 (35.3%)	20 (58.8%)
Presence of obstructive sign (*n* (%))	8 (47.1%)	2 (11.8%)	10 (29.4%)
Location (*n* (%))			
Jejunum	11 (64.7%)	5 (29.4%)	16 (47.1%)
Ileum	6 (35.3%)	11 (64.7%)	17 (50.0%)
Indeterminate	0	1 (5.9%)	1 (2.9%)
Route of enteroscopy (*n* (%))			
Anterograde	13 (76.5%)	8 (47.1%)	21 (61.8%)
Retrograde	3 (17.6%)	7 (41.2%)	10 (29.4%)
Bidirectional	1 (5.9%)	2 (11.8%)	3 (8.8%)
Types of enteroscopy (*n* (%))			
Double-balloon	12 (70.6%)	14 (82.4%)	26 (76.5%)
Single-balloon	5 (29.4%)	3 (17.6%)	8 (23.5%)
Procedure time (minutes), median (range)	70 (20-250)	83 (29-234)	75 (20-250)
Balloon dilation (*n* (%))	2 (11.8%)	4 (23.5%)	6 (17.6%)
Procedure-related complications (*n* (%))	0	2 (11.8%)	2 (5.9%)
Perforation	0	1 (5.9%)	1 (2.9%)
Pancreatitis	0	1 (5.9%)	1 (2.9%)

BAE: balloon-assisted enteroscopy; NSAID: nonsteroidal anti-inflammatory drug.

**Table 2:** Association between clinical characteristics and successful retrieval of all foreign bodies and capsule endoscopes using balloon-assisted enteroscopy.

Variables	Overall FB			Capsule endoscopes		
	Retrieval success (*n* = 17)	Retrieval failure (*n* = 17)	*P*	Retrieval success (*n* = 7)	Retrieval failure (*n* = 11)	*P*
Age (yr), median (range)	62 (32-92)	55 (19-86)	0.389	44 (33-70)	42 (24-86)	0.717
Sex (*n* (%))			0.714			0.596
Male	11 (64.7%)	12 (70.6%)		6 (85.7%)	7 (63.6%)	
Female	6 (35.3%)	5 (29.4%)		1 (14.3%)	4 (36.4%)	
Previous abdominal operation history (*n* (%))	7 (41.2%)	9 (52.9%)	0.492	0	4 (36.4%)	0.119
Previous small bowel operation (*n* (%))	3 (17.6%)	7 (41.2%)	0.259	0	3 (27.3%)	0.245
FB (capsule or not) (*n* (%))			0.169			NA
Capsule	7 (41.2%)	11 (64.7%)				
Not capsule	10 (58.8%)	6 (35.3%)				
Category of FB (*n* (%))			0.695			NA
Capsule endoscope	7 (41.2%)	11 (64.7%)				
Duodenal stent	3 (17.6%)	2 (11.8%)				
Biliary drainage catheter	3 (17.6%)	1 (5.9%)				
Gallstones	2 (11.8%)	1 (5.9%)				
Other	2 (11.8%)^†^	2 (11.8%)^‡^				
FB shape (*n* (%))			0.748			NA
Blunt	10 (58.8%)	12 (70.6%)				
Long	6 (35.3%)	4 (23.5%)				
Sharp-pointed	1 (5.9%)	1 (5.9%)				
Length of FB (mm), median (range)	26 (10-120)	26 (20-220)	0.614			NA
Diameter of FB (mm), median (range)	11 (1-50)	11 (1-20)	0.808			NA
Duration of retention (days), median (range)	8 (1-23)	15 (0-364)	0.230	8 (1-22)	14 (1-61)	0.126
FB location (*n* (%))			0.094			0.203
Jejunum	11 (64.7%)	5 (29.4%)		3 (42.9%)	1 (9.1%)	
Ileum	6 (35.3%)	11 (64.7%)		4 (57.1%)	9 (81.8%)	
Indefinite	0	1 (5.9%)		0	1 (9.1%)	
Symptom presence (*n* (%))	14 (82.4%)	6 (35.3%)	0.013	5 (71.4%)	1 (9.1%)	0.013
Obstructive sign (*n* (%))	8 (47.1%)	2 (11.8%)	0.057	5 (71.4%)	9 (81.8%)	1.000
Enteroscopy type (*n* (%))			0.688			1.000
Double-balloon	12 (70.6%)	14 (82.4%)		5 (71.4%)	9 (81.8%)	
Single-balloon	5 (29.4%)	3 (17.6%)		2 (28.6%)	2 (18.2%)	
Enteroscopy route (*n* (%))			0.210			0.230
Anterograde	13 (76.5%)	8 (47.1%)		5 (71.4%)	4 (36.4%)	
Retrograde	3 (17.6%)	7 (41.2%)		1 (14.3%)	6 (54.5%)	
Bidirectional	1 (5.9%)	2 (11.8%)		1 (14.3%)	1 (9.1%)	
Balloon dilation (*n* (%))	2 (11.8%)	4 (23.5%)	0.656	2 (28.6%)	3 (27.3%)	1.000

FB: foreign body; CE: capsule endoscope; BAE: balloon-assisted enteroscopy; NA: not applicable. ^†^Other, needle and intragastric bariatric balloon. ^‡^Other, embolization coil and razor blade.

**Table 3:** Univariable and multivariable analyses of factors associated with successful retrieval of all foreign bodies using balloon-assisted enteroscopy.

Variables	Univariable analysis			Multivariable analysis		
	OR	95% CI	*P*	OR	95% CI	*P*
Age (per 1 year increase)	1.02	0.98-1.05	0.421	1.01	0.96-1.07	0.619
Sex (male vs. female)	0.76	0.18-3.23	0.714	1.82	0.16-20.49	0.628
Previous abdominal operation history	0.62	0.16-2.42	0.493			
Previous small bowel operation	0.31	0.06-1.48	0.141	0.09	0.01-1.37	0.082
FB (not capsule vs. capsule)	2.62	0.66-10.48	0.174	0.79	0.06-9.88	0.853
FB shape			0.750			
Blunt	1.00					
Long	1.80	0.39-8.22	0.448			
Sharp-pointed	1.20	0.07-21.72	0.902			
Length of FB (per 1 mm increase)	1.00	0.98-1.02	0.970			
Diameter of FB (per 1 mm increase)	1.06	0.96-1.17	0.259			
Duration of retention (per 1 day increase)	0.95	0.88-1.02	0.178	0.94	0.83-1.06	0.314
FB location			0.170			0.577
Jejunum	1.00			1.00		
Ileum	0.25	0.06-1.06	0.060	0.55	0.07-4.52	
Indefinite	NA	NA	NA	NA	NA	NA
Symptom (presence vs. absence)	8.56	1.74-42.17	0.008	13.40	1.10-162.56	0.042
Obstructive sign (presence vs. absence)	6.67	1.15-38.60	0.034	2.45	0.30-20.18	0.405
Enteroscopy type (SBE vs. DBE)	1.94	0.38-9.88	0.423			
Enteroscopy route			0.224			
Anterograde	1.00					
Retrograde	0.26	0.05-1.33	0.106			
Bidirectional	0.31	0.02-3.97	0.366			
Balloon dilation (done vs. not done)	0.43	0.07-2.76	0.376			

OR: odds ratio; CI: confidence interval; FB: foreign body; NA: not applicable; SBE: single-balloon enteroscopy; DBE: double-balloon enteroscopy.

**Table 4:** Univariable analyses of factors associated with successful retrieval of capsule endoscopes using balloon-assisted enteroscopy.

Variables	Univariable analysis		
	OR	95% CI	*P*
Age (per 1 year increase)	1.00	0.96-1.05	0.965
Sex (male vs. female)	3.43	0.30-39.64	0.324
Duration of retention (per 1 day increase)	0.92	0.82-1.04	0.183
CE location			
Jejunum	1.00		
Ileum	0.15	0.01-1.90	0.142
Symptoms (presence vs. absence)	25.00	1.80-346.69	0.016
Obstructive sign	0.75	0.06-10.23	0.829
Enteroscopy type (SBE vs. DBE)	1.80	0.19-16.98	0.608
Enteroscopy route			0.278
Anterograde	1.00		
Retrograde	0.13	0.01-1.61	0.113
Bidirectional	0.80	0.04-17.20	0.887
Balloon dilation (done vs. not done)	1.07	0.13-8.79	0.952

OR: odds ratio; CI: confidence interval; CE: capsule endoscope; NA: not applicable; SBE: single-balloon enteroscopy; DBE: double-balloon enteroscopy.

## Data Availability

The retrospective data used to support the findings of this study are included within the article. Access to raw data is restricted.

## Abbreviations

KASID: Korean Association for the Study of Intestinal Diseases
BAE: Balloon-assisted enteroscopy
FB: Foreign body
CE: Capsule endoscope
SEMS: Self-expandable metal stent
DBE: Double-balloon enteroscopy
SBE: Single-balloon enteroscopy
CT: Computed tomography
OR: Odds ratio
CI: Confidence interval
NSAID: Nonsteroidal anti-inflammatory drug
NA: Not applicable.
